# The carboxypeptidase D homolog *silver* regulates memory formation via insulin pathway in *Drosophila*

**DOI:** 10.1007/s13238-016-0291-4

**Published:** 2016-07-18

**Authors:** Binyan Lu, Yi Zhao, Jie Zhao, Xiaoyang Yao, Yichun Shuai, Weiwei Ma, Yi Zhong

**Affiliations:** 1 McGovern Institute for Brain Research, Ministry of Education Key Laboratory of Protein Sciences, Tsinghua-Peking Center for Life Sciences, School of Life Sciences, Tsinghua University, Beijing, 100084 China; 2 State Key Laboratory of Systematic and Evolutionary Botany, Institute of Botany, Chinese Academy of Sciences, Beijing, 100093 China

**Dear Editor,**

What is the molecular basis of memory formation? Many genes have been implicated in this process, including those involved in neural cell adhesion, mRNA transport, translation control, and cAMP-PKA signaling. *Drosophila*, with easy accessibility to genetic, molecular, and behavioral analyses, was also employed in olfactory learning studies and many key genes underlying memory formation were identified in these studies (Bellen et al., 2010). The majority of identified genes have been shown to be intensively expressed in the mushroom body (MB) of the *Drosophila* brain (Davis, 2005), including *rut* (adenylyl cyclase), *DCO* (the catalytic subunit of PKA), and *AKAP Yu* (Heisenberg, 2003; Davis, 2005; Lu et al., 2007). However, these genes are far from enough to understand memory formation comprehensively.

In order to identify more genes involved in memory formation, we previously generated 2,667 enhancer trap mutants, each of which contains a p-element (P{GawB}) insertion (Liu et al., 2008). The insertion includes a Gal4 sequence that can be used to label the expression pattern of the disrupted gene and 368 mutants were selected because their perturbed-gene expression enriched in the mushroom body. These selected mutants were then screened for 3 h memory performance in a well-defined olfactory conditioning paradigm, and one strain (No. 1021) was found defective. Plasmid rescue showed that the p-element of this mutant located in the first intron of the gene *silver* (*svr*) (Fig. 1A). Western blot analysis revealed that protein expression level of Svr was significantly decreased in this mutant (*svr*^*1021*^, Fig. 1B).

**Figure 1:**
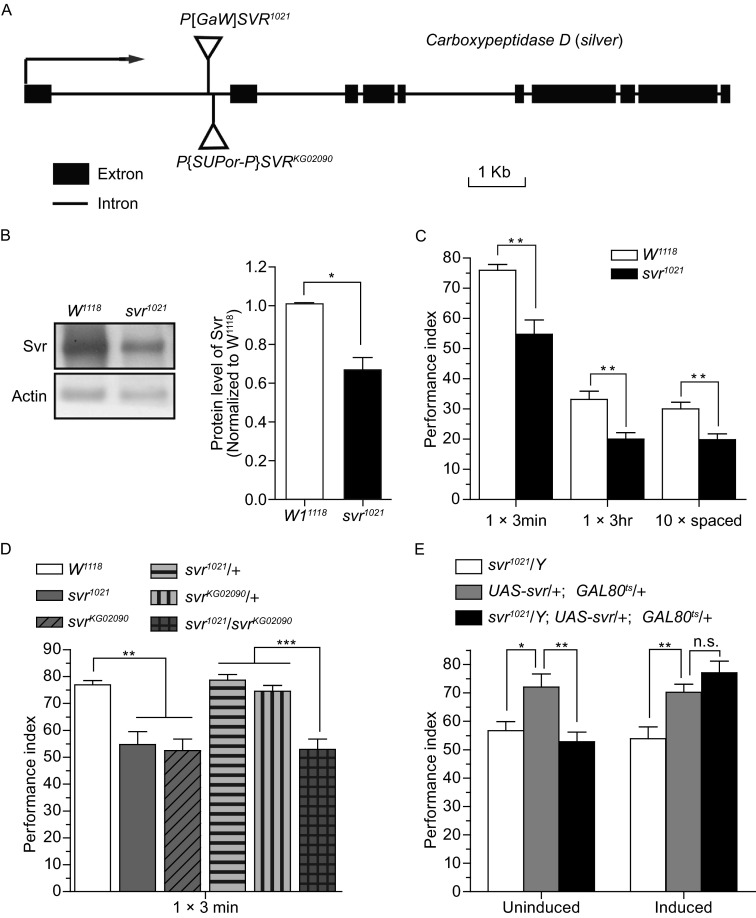
**The memory formation of** ***svr*** ^***1021***^ **mutant is impaired and acute expression of** ***svr*** ^**+**^ **transgene in the** ***svr*** ^***1021***^ **mutant can rescue the impairment**. (A) P element insertion sites of the *svr* gene in *svr* ^*1021*^ and *svr* ^*KG02090*^ alleles. Boxes indicate exons and the lines represent introns. (B) Western blot analysis showed that Svr expression was greatly reduced in *svr* ^*1021*^ mutant (*t*-test, *P* < 0.05). *n* = 3 (C). Memory retention at 3 min and 3 h after one-cycle training and 24 h memory after spaced training were significantly decreased in *svr* ^*1021*^ mutant (*t*-test, *P* = 0.001, 0.004, 0.005 compared to *w* ^*1118*^ for 3 min, 3 h, 24 h after spaced training, respectively). *n* = 6–8. (D) Genetic complementation analysis established that disruption the *svr* gene was responsible for the memory defect. The 3-min memory in double-heterozygote (*svr* ^*1021*^/*svr* ^*KG02090*^) was significantly lower than the other two heterozygous flies (*svr* ^*1021*^/+ or *svr* ^*KG02090*^/+) or the control flies (ANOVA, *P* < 0.001). *n* = 6–8. (E) The 3-min memory defect in *svr* mutant was reversed by restoration of expression in *svr* ^*1021*^ labeled neuron after 3 day heat shock (ANOVA, Induced: *P* = 0.008 for *svr* ^*1021*^/*Y* compared to *UAS-svr*/+; *GAL80* ^*ts*^/+, *P* = 0.387 for *svr* ^*1021*^/*Y*; *UAS-svr*/+; *GAL80* ^*ts*^/+ compared to *UAS-svr*/+; *GAL80* ^*ts*^/+; Uninduced, *P* = 0.013 for *svr* ^*1021*^/*Y* compared to *UAS-svr*/+; *GAL80* ^*ts*^/+, *P* = 0.003 for *svr* ^*1021*^/*Y*; *UAS-svr*/+; *GAL80* ^*ts*^/+ compared to *UAS-svr*/+; *GAL80* ^*ts*^/+). *n* = 6–7. Data are shown as the mean ± SEM *P < 0.05; **P < 0.01; ***P < 0.001

Svr has been reported to involve in viability and behaviors such as cold and ethanol sensitivity, as well as long-term memory in courtship behavior (Sidyelyeva et al., 2010). To explore the function of Svr in olfactory memory formation, we tested the performance index of *svr*^*1021*^ in different time points after training. Memory of the *svr* mutant (*svr*^*1021*^) exhibited a significant impairment at both 3 min and 3 h after one-cycle training (Fig. 1C). And 24 h memory of the mutant was also disrupted in *svr*^*1021*^ mutant after spaced training (Fig. 1C). The memory defect was unlikely to be caused by deficiency in sensorimotor system since no abnormality in shock or odor avoidance was observed (Table S1). Finally, in order to confirm that the 3 min memory defect is not a result of random background mutation, we conducted a genetic complementation experiment, using *svr*^*1021*^ and another independent P-element insertion allele, *svr*^*KG02090*^. Indeed, although both heterozygote mutants (*svr*^*1021*^/*+* and *svr*^*KG02090*^/*+*) had normal 3 min memory, such memory was impaired in double-heterozygote mutant (*svr*^*1021*^/*svr*^*KG02090*^) (Fig. 1D).

One previous study showed that *svr* is involved in *Drosophila* development (Sidyelyeva et al., 2010). To figure out whether the disrupted memory formation of *silver* mutant resulted from the abnormal development or the interference of physiological process of neural system, we acutely manipulated the expression of *svr*^*+*^ transgene in adult flies with the TARGET system. In this system, the Gal4-induced expression is suppressed by a ubiquitously expressed Gal80^ts^ protein at the permissive temperature (18°C), but not at the restrictive temperature (30°C). The gene *svr* contains three carboxypeptidase domains, and has five endogenous transcriptional forms as a result of alternative splicing (Sidyelyeva et al., 2010). Previous findings suggested that the functions of CPD domain 1 and 2 are largely redundant, and the inactive CPD domain 3 is required for fully rescuing the mutant’s phenotype (Sidyelyeva et al., 2010). As a result, we used a longer form containing all three CPD domains (UAS::svr1B-2-3-t1 construct) to restore memory (Sidyelyeva et al., 2010). Acute expression of *svr* transgene in *svr*-labeled-neurons of *svr* mutant flies (*svr*^*1021*^/*Y*; *UAS-svr*/+; *Gal80*^*ts*^/+) effectively rescued the perturbed memory in *svr* mutant to a comparable level in the control group (*UAS-svr*/+; *Gal80*^*ts*^/+) (left panel, Fig. 1E). On the other hand, no significant difference between svr acute expression group (*svr*^*1021*^/*Y*; *UAS-svr*/+; *Gal80*^*ts*^/+) and svr mutant group (*svr*^*1021*^/*Y*) was detected in un-induced conditions (right panel, Fig. 1E). These results suggest that Svr interferes the physiological process of memory formation.

Next, in order to find out the functioning neural circuit of Svr in fly brain, we visualized the Gal4 expression pattern of *svr*^*1021*^ using GFP labeling. Confocal imaging of the GFP signal in *svr*^*1021*^/+; *UAS-mCD8*::*GFP*/+ revealed a preferential expression in two major compartments: the MB and a small group of neurosecretory cells located in the dorsal/medial region of the fly brain (Fig. 2A and 2B). Other *svr* enhancer trap lines *svr*^*NP2073*^ and *svr*^*NP3600*^ showed similar expression patterns to *svr*^*1021*^ (Fig. 2A). And the memory formation of these svr-GAL4 mutants was also impaired (Fig. S1). Considering the fact that insulin-producing cells (IPCs) overlap with this cluster of neurosecretory cells (Nässel et al., 2013), we proposed that both the MB and IPC could be candidate brain areas where *svr* affects memory formation. The GAL4-UAS binary system was used to specifically express *svr*^+^ transgene in these cells of interest. We took the OK107-Gal4 to cover all mushroom body neurons, and dilp2-Gal4 to cover the IPCs (Nässel et al., 2013). Subsequent behavioral assays showed that specific expression of *svr* in the IPCs (*svr*^*KG02090*^/*Y*; *UAS-svr*/+; *dilp2-Gal4*/+) rescued the memory deficiency in *svr* mutant (*svr*^*KG02090*^/*Y*; *UAS-svr*/+, Fig. 2D). However, *svr* expression in the MB (*svr*^*KG02090*^/*Y*; *UAS-svr*/+; *OK107-Gal4*/+), was not able to generate similar rescuing effect (Fig. 2C). Thus, to our surprise, IPCs rather than MB are the crucial region where Svr influences memory formation.

**Figure 2:**
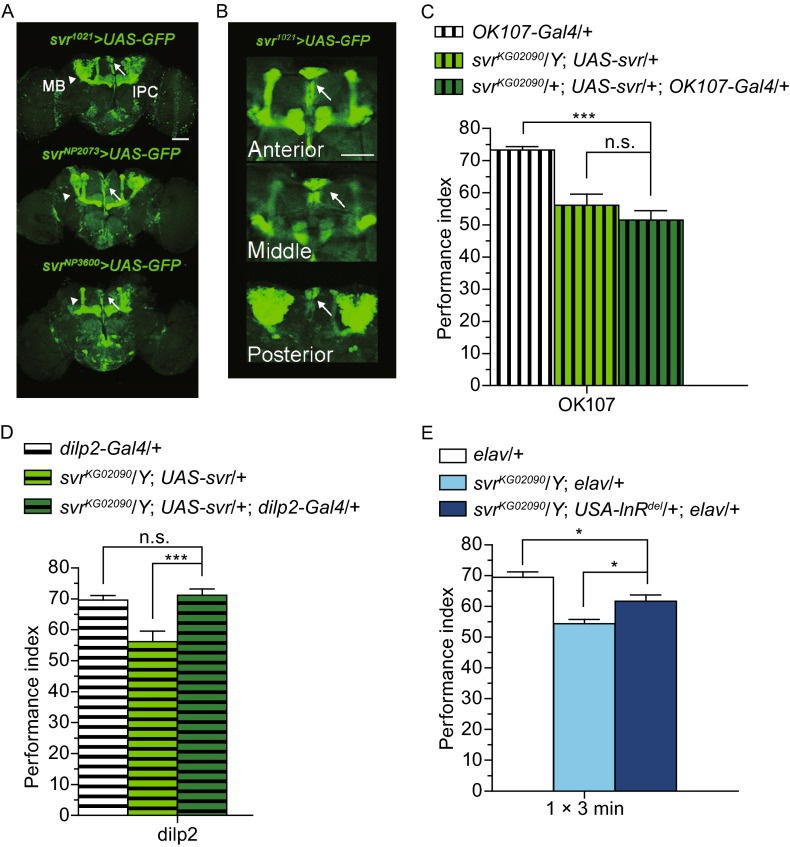
**Restricted expression of** ***svr*** ^**+**^ **in the insulin-producing cells is sufficient to rescue the** ***svr*** **memory formation defect and insulin signaling is involved in** ***svr*** **regulated memory formation**. (A) The confocal imaging of three types of *svr-Gal4* driven GFP expression in the adult brain revealed preferential expression in the MB and clusters of median neurosecretory cells. Scale bar is 100 μm. (B) Enlarged views of anterior, middle, and posterior section of *svr* ^*1021*^ *-Gal4* driven GFP expression. Arrow indicates IPCs. Scale bar is 50 μm. (C and D) Immediate memory defect in *svr* ^*KG02090*^ mutant was rescued by inducing *svr* ^+^ transgene in IPCs (*dilp-Gal4* driven) (ANOVA, *P* = 0.0007 for *svr* ^*KG02090*^/*Y*; *UAS-svr*/+; *dilp2-Gal4*/+ compared to *svr* ^*KG02090*^/*Y*; *UAS-svr*/+, *P* = 0.857 for *svr* ^*KG02090*^/*Y*; *UAS-svr*/+; *dilp2-Gal4*/+ compared to *dilp2-Gal4*/+), but not by inducing *svr* ^*+*^ transgene in MB (*OK107-Gal4* driven) (ANOVA, *P* = 0.426 for *svr* ^*KG02090*^/*Y*; *UAS-svr*/+; *OK107-Gal4*/+ compared to *svr* ^*KG02090*^/*Y*; *UAS-svr*/+, *P* < 0.0001 for *svr* ^*KG02090*^/*Y*; *UAS-svr*/+; *OK107-Gal4*/+ compared to *OK107-Gal4*/+). *n* = 6–7. For the X chromosome-located *svr* mutant, only male flies results were shown. (E) Expressing constitutively active InR (*UAS-InR* ^*del*^) in nervous system partially reversed the memory impairment in *svr* ^*KG02090*^ (ANOVA, *P* = 0.016 for *elav*/+ compared to *svr* ^*KG02090*^/*Y*; *UAS-InR* ^*del*^/+; *elav*/+, *P* = 0.023 for *svr* ^*KG02090*^/*Y*; *elav*/+ compared to *svr* ^*KG02090*^/*Y*; *UAS-InR* ^*del*^/+; *elav*/+). *n* = 6–7. Data are shown as the mean ± SEM. *P < 0.05; **P < 0.01; ***P < 0.001

*Drosophila* IPCs are functionally similar to mammalian pancreatic islet β cells, because they produce several kinds of *Drosophila* insulin-like peptides (dilp1-dilp8), which have analogous functions to insulin (Nässel et al., 2013). Apart from the modulation of energy homeostasis, insulin and its receptors also participate in cognitive processes in the central nervous system (Babri et al., 2007). Previous studies showed that in both rats and humans, appropriate increase of insulin in specific brain areas can improve certain cognitive abilities, such as spatial memory (Benedict et al., 2004; Babri et al., 2007). In addition, in *C. elegans*, insulin/IGF-1 receptor mutant *daf-2* has augmented short-term and long-term memory performance in early adulthood (Kauffman et al., 2010). All these findings suggest the participation of insulin pathway in memory formation, although the responsible mechanism has not been clarified. In the present study, Svr functioning in a restricted group of IPCs—dilp2 neurons to regulate memory formation implies that the potential connection between Svr and insulin pathway may be involved in memory formation (Fig. 2D).

*Silver* encodes the homolog of human carboxypeptidase D (CPD) in *Drosophila*. Carboxypeptidases and endopeptidases can turn precursors into peptides (Fricker, 2005). CPD is a member of the carboxypeptidase family and has a wide range of substrates, including growth factors, hormones, and neuropeptides (Skidgel and Erdos, 1998). Another member of the carboxypeptidase family, carboxypeptidase E (CPE) has been reported to cause proinsulin processing defect in its mice mutant (Naggert et al., 1995). Based on the fact that CPD and CPE share similar enzymatic properties and comparable distribution in the rat central neural system, CPD is speculated to be functionally redundant with CPE (Dong et al., 1999). Consequently, insulin processing is a potential substrate pathway of *svr*. The *Drosophila* insulin/insulin-like growth factor signaling (IIS) system is similar to its human counterpart. It comprises a single insulin receptor (InR) that mediates the function of all eight insulin-like peptides (ILPs), from dilp1 to dilp8 (Nässel et al., 2013). InRs are expressed ubiquitously, but the eight ILPs are expressed in specific tissues, presumably in response to different inputs. Therefore, we overexpressed a constitutively active InR to increase the insulin signal in the pan-neural system of the *svr* mutant. We found that the expression of constitutively active InR (*svr*^*KG02090*^/*Y*; *UAS-InR*^*del*^/+; *elav*/+) partially rescued the memory impairment in *svr* mutant (*svr*^*KG02090*^, Fig. 2E).

All our findings suggest that Svr regulates the memory formation via insulin pathway in neurosecretory cells outside MB.

## Electronic supplementary material

Below is the link to the electronic supplementary material.

Supplementary material 1 (PDF 120 kb)
