# Supplementary material for: The carboxypeptidase D homolog silver regulates memory formation via insulin pathway in Drosophila
Source: Protein Cell. 2016 Jul 18;7(8):606–10. doi: 10.1007/s13238-016-0291-4 (PMC4980332; doi:10.1007/s13238-016-0291-4)
Supplement: Supplementary file 1 — Supplementary material 1 (PDF 120 kb) [file 13238_2016_291_MOESM1_ESM.pdf]

Supplemental Materials

Materials and Methods

Isolation of memory-defective mutants

Transposon mutagenesis was performed as described previously (Dubnau et al., 2003; Liu et al., 2008). 2,667 homozygous strains were subjected to expression pattern analysis. 368 lines specifically expressed in the MB were considered as candidates for screening 3hr memory defect after one-cycle training. Based on molecular, genetic and behavioral characteristics, we identified a *svr* mutant line，svr^{1021}， which had memory defect.

Fly stocks

All flies were reared at 25°C and 60% humidity on standard cornmeal medium with 12/12 hr light/dark cycle. The $svr^{KG02090}$ and $UAS\text{-}InR^{del}$ (expressed a constitutively active InR with most of its α subunit deleted) lines were obtained from Bloomington Stock Center (Bloomington, IN). *UAS::svr1B-2-3-t1* (termed as *UAS*-*svr*) transgenic fly was kindly provided by Dr. Lloyd D. Fricker. *dilp2-GAL4* was a gift from Dr. Ping Shen. The *OK107*, *elav-Gal4* and $tubulin\text{-}Gal80^{ts}$ ($Gal80^{ts}$) lines were stocks in our lab. To eliminate genetic background differences, all strains used in behavioral experiments were outcrossed with w^{1118} *(isoCJ1)* wild-type flies for at least five generations.

Plasmid rescue of P-element insertion

Plasmid rescue was carried out by digesting total genomic DNA from svr^{1021} mutant with either EcoRI or XbaI, ligating the resulting restriction fragments and then transforming isolated ampicillin-resistance plasmids to E.coli. PCR. Sequencing of genomic DNA flanking and genomic blast confirmed the insertion site of P{GawB} transposon.

Pavlovian olfactory aversive conditioning

Training and test were performed at 25°C and 70% relative humidity as described previously (Tully et al., 1994). During one-cycle training, around 100 flies were sequentially exposed to two aversive odors, 3-octanol (OCT) (Fluka, 1.5×10^{-3} diluted in heavy mineral oil) and 4-methylcycloohexanol (MCH) (Fluka, 1×10^{-3} diluted in heavy mineral oil) for 60s with 45s flush of fresh air after each odor. Flies received electric foot shock (twelve 1.5s pulse of 60V) during the presence of the first odor (CS+) but not the second (CS-). In spaced training, flies were exposed to ten training sessions with 15 min rest intervals.

During test, trained flies were allowed to choose between CS+ and CS- in a T-maze for 120s. A performance index (PI) was calculated from the distribution of flies in the two T-maze arms. It was the result of the difference between fly numbers in the two T-maze arms divided by the total fly number of the two arms. To eliminate odor bias, each experiment (n = 1) consisted of two reciprocal groups, with one trained to associate OCT with shock and the other to associate MCH with shock. The final PI was the average of PIs from the two groups.

Sensorimotor responses

To test the olfactory acuity, odor-avoidance responses were quantified by exposing groups of about 100 untrained flies to the test odor (either OCT or MCH, 1.5×10-3 and 1×10-3 dilution, respectively) versus fresh air in the T-maze. To test the shock reactivity, groups of 100 untrained flies were exposed to two T-maze arms with 60V electric foot shock delivered to one of the arms but not the other. In both tests, flies were allowed to make a choice between the two arms for 120s and PI was calculated as described in the memory test.

Heat shock regimen

Flies carrying the *tubulin-Gal80^{ts}* were raised and maintained in 18°C to minimize any potential "leaky" expression during development. 2-5 days old progenies were collected and divided into two groups. The induced group was transferred to a 30°C incubator for 3 days, whereas the uninduced control group was kept at 18°C. Both

groups were allowed to recover at 25°C for at least 1hr before behavioral experiments.

Generation of antibody and western blot

The anti-Svr antibody was generated by injecting a purified polypeptide fragment (DRLEQSHVHQLR) into rabbits. Subsequently the antiserum was affinity-purified by Protein A spin chromatography kit (Pierce, Rockford, IL). For Western blot, approximately five adult brains of each genotype were collected in a microcentrifuge tube. Each brain sample was homogenized with 25 µl working loading buffer in an ice bath using a pellet pestle. Immediately after heating the homogenate for 10 min at 70°C, the debris was spun down and 20 µl supernatant was loaded into 3-8% TA gel (Invitrogen) and blotted to a nitrocellulose membrane (Millipore). The blot was probed with primary antibodies against Svr (1:200) and Actin (1:4,000) (Zhongshan Gold Bridge Biotech) overnight at 4 °C and then with HRP-conjugated goat anti-rabbit IgG (Zhongshan Gold Bridge Biotech) for 2 h at room temperature. Finally, the exposed films were scanned and analyzed by Image J software (NIH).

Confocal imaging

5 days old adult brains were dissected in PBS, transferred to fixative buffer (4% paraformaldehyde in PBS), and vacuumed for 30 min at room temperature. For GFP expression detection, the fixed and vacuumed brain was mounted in FocusClear (Pacgen Biopharmaceuticals). The brains were imaged on a Zeiss 710 laser scanning confocal microscope. Image stacks was obtained with Imaris 7.0 software (Bitplane, Zurich, Switzerland).

Statistics

All data were presented as means ± SEM and analyzed by Student's t tests or ANOVA followed by Sidak's post hoc test in Graphpad Prism (Graphpad Software, Inc.). Asterisks indicate statistically critical value (*$p < 0.05$; **$p < 0.01$; ***$p < 0.001$); 'n.s.' denotes no significant difference ($p > 0.05$).

Table S1. Sensorimotor responses to odors and foot-shock.

Genotype	Olfactory acuity		Shock avoidance
	OCT (1.5×10^{-3})	MCH (1×10^{-3})	(60V)
W^{1118}*(isoCJ1)*	36.0±3.1	39.9±2.6	65.2±3.4
svr^{1021}	39.5±2.3	40.8±3.5	64.3±1.6
$svr^{KG02090}$	35.6 ± 3.4	36.5 ± 2.1	67.8 ± 3.6

[a] Flies were challenged with 60V shock versus no shock, or an odor versus fresh air and were required to make a binary choice.

[b] No significant difference was detected between *svr* mutants and control group ($p>0.526$, $n \geq 6$, ANOVA).

Figure S1. The memory formation was impaired in the svr-GAL4 mutant.

Memory retention at 3 minutes after one-cycle training were significantly decreased in svr-GAL4 mutant svr^{NP3600} (t-test, p=0.0008 compared to w^{1118}). n=10-12.
